# The safety of Canadian rural maternity services: a multi-jurisdictional cohort analysis

**DOI:** 10.1186/s12913-015-1034-6

**Published:** 2015-09-23

**Authors:** Stefan Grzybowski, John Fahey, Barbara Lai, Sharon Zhang, Nancy Aelicks, Brenda M. Leung, Kathrin Stoll, Rebecca Attenborough

**Affiliations:** University of British Columbia, 300-5950 University Blvd, Vancouver, BC V6T 1Z3 Canada; Reproductive Care Program of Nova Scotia, Suite 700-5991 Spring Garden Rd, Halifax, NS B3H 1Y6 Canada; Alberta Perinatal Health Program, Alberta Health Services, 10030-107 Street NW Edmonton, Alberta, T5J 3E4 Canada; School of Population & Public Health, UBC, Vancouver, Canada

## Abstract

**Background:**

Small Canadian rural maternity services are struggling to maintain core staffing and remain open. Existing evidence states that having to travel to access maternity services is associated with adverse outcomes. The goal of this study is to systematically examine rural maternal and newborn outcomes across three Canadian provinces.

**Methods:**

We analyzed maternal newborn outcomes data through provincial perinatal registries in British Columbia, Alberta and Nova Scotia for deliveries that occurred between April 1st 2003 and March 31st 2008. All births were allocated to maternity service catchments based on the residence of the mothers. Individual catchments were stratified to service levels based on distance to access intrapartum maternity services or the model of maternity services available in the community. The amalgamation of analyses from each jurisdiction involved comparison of logistic regression effect estimates.

**Results:**

The number of singleton births included in the study is 150,797. Perinatal mortality is highest in communities that are greater than 4 h from maternity services overall. Rates of prematurity at less than 37 weeks gestation are higher for rural women without local access to services. Caesarean section rates are highest in communities served by general surgical models.

**Conclusion:**

Composite analysis of data from three Canadian provinces provides the strongest evidence to date demonstrating that we need to sustain small community maternity services with and without caesarean section capability.

## Background

Rural and remote Canadian communities typically have populations of less than 10,000 people. Most rural communities of greater than 5000 people have a hospital which includes in-patient beds, an emergency department, and may offer maternity services and surgical services. During the past 15 years there has been a significant erosion of small maternity services across rural Canada [1–4]. In British Columbia alone there have been 20 closures since 2000 [5]. Closures have occurred for a number of reasons including difficulty recruiting maternity care providers [6–10] concerns about the quality of maternal and newborn outcomes in small facilities [11, 12] and an emphasis on regionalization and consequent centralization of rural services which has swept across Canada [13–15]. A comprehensive review of policy at both provincial and national levels highlights the lack of a systematic approach to planning rural maternity service delivery [16]. The safety of small rural maternity services with and without caesarean section is not well researched, though on balance the evidence suggests that even without local caesarean section capacity, small maternity services can provide good care and that outcomes are likely improved if there is a limited local service rather than no local intrapartum service at all [7, 11, 17, 18].

A number of authors have published studies examining the safety of individual rural hospitals both with and without caesarean section capabilities [19–21]. These have generally demonstrated positive results, but are potentially subject to publication bias as the motivation to disseminate results usually is driven from a positive perspective. Other authors have looked at multiple rural sites and larger numbers of births to show that service models with and without caesarean section at rural sites are associated with good outcomes [22]. There have also been national and regional studies examining outcomes that have showed positive findings for small rural services [23, 24].

A provincial rural study using geographically defined population catchments surrounding maternity services examined the relationship between level of access to services and outcomes [25]. This evidence has shown that distance to services is related to adverse maternal and newborn outcomes [25]. Specifically, the odds of experiencing perinatal mortality for women in British Columbia who live more than 4 h away from maternity services were 3.17 times higher than women served by local obstetricians. Women living 2 to 4 h away from services had a higher induction rate. The odds of an unplanned out-of-hospital delivery were six times higher among women living 1 to 2 h away from services. Overall, women who had local access to maternity services, even without caesarean section had outcomes similar to women resident in catchments served by obstetricians. Local access to caesarean section dramatically increased the proportion of women able to deliver at their local hospital (30 % if no local surgical services, vs. >75 % with local surgical services) [26]. Previous work done in Alberta by Iglesias *et al.* showed a similar effect associated with the presence or absence of local surgical services in a rural community though the rates were 22.1 % and 70.1 %, respectively [22].

The objective of this multi-jurisdictional analysis is to examine the safety of rural Canadian maternity services stratified by service delivery level across three Canadian provinces.

## Methods

We accessed maternal newborn outcomes data through provincial perinatal registries in British Columbia, Alberta and Nova Scotia for deliveries that occurred between April 1st 2003 and March 31st 2008. We focused our attention on non-metropolitan birth data and consequently excluded: southern Vancouver Island (Victoria and environs), Vancouver, the lower mainland, and the Fraser Valley from the BC data; Edmonton, Calgary and surrounding areas from the Alberta data; and, Halifax from the Nova Scotia data. We also excluded multiple births and infants born with congenital anomalies from the data set.

The residence location of rural women in BC and Alberta is defined by postal code, not street address. In Nova Scotia, geocoding was done using a combination of street address, community name, municipality code and postal code (in order of decreasing accuracy) for all rural residents. Consequently in BC and Alberta, we used centroids of rural postal codes to geographically define the population catchments surrounding each rural facility in each province and also the distance women needed to travel to access maternity services when no local hospital services were available [27]. In Nova Scotia, we used the geocoded location and calculated actual travel time to the nearest facility. Surface travel time by road was used to create the 1 hour catchments around each rural facility as well as cohorts of rural women who had to travel 1–2 h, 2–4 h, and more than 4 h to access the nearest maternity services in BC and Alberta (Table 1) [22, 25–27]. As Nova Scotia is a smaller province which is more densely populated than Alberta or British Columbia, some of the service levels were not present. We cross-checked distances defined using a GIS approach with Google Maps® functionality in a selection of communities to strengthen accuracy in definition of catchments, whereas every residence-to-facility driving time was obtained programmatically from Google Maps® for the smaller Nova Scotia dataset [28] .

**Table 1:** Description of maternity service levels

Service Level	Description
1	Nearest maternity services greater than 4 h away
2	Nearest maternity services within 2 to 4 h
3	Nearest maternity services within 1 to 2 h
4	Primary care maternity services (without local surgical care)
5	Maternity services provided by General Practitioner(s) with Enhanced Surgical Skills (GPESS(s))
6	Maternity services provided by a mixed model (GPESS(s) & specialist surgeon(s))
7	Maternity services provided by general surgeons
8	Maternity services provided by obstetricians

Service level was defined for each rural hospital providing intrapartum services using perinatal services data in each province, cross-checked by phone inquiry with local administration when there were changes to the service during the time frame of this study or the service model was uncertain (Table 1). When changes in service level occurred, we assigned catchment data to the appropriate service level by year.

One of the challenges of this multi-jurisdictional analysis is that currently, there is no formal data-sharing agreement between provincial database registries in Canada, nor are there pan-Canadian standards for perinatal data elements collected [29] . We chose to include data from British Columbia, Alberta and Nova Scotia as the perinatal databases in these jurisdictions have similar characteristics. This allowed parallel investigation and eventual amalgamation of results. We included all data elements that were clinically significant and met the following criteria: (1) availability of common fields; (2) consistency in operationalization of terms; (3) consistency in data collection methods; and, (4) congruence in original purpose for data collection.

**Fig. 1:**
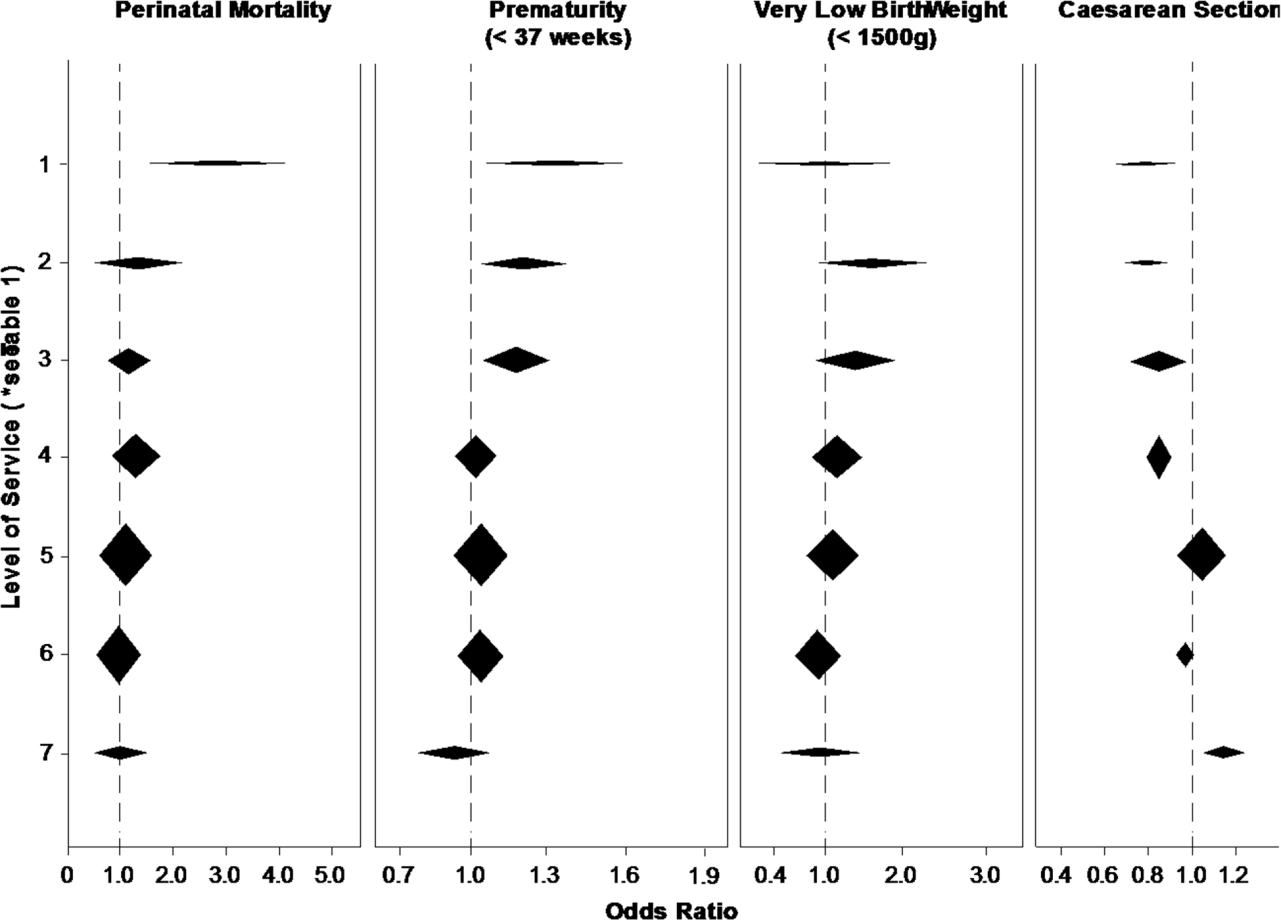
Composite forest plots demonstrating outcomes related to level of service

Analysis was conducted in a parallel fashion in each jurisdiction. Adjustments were made for maternal age, parity, prior neonatal death, previous caesarian section, prior still birth, diabetes, and hypertension. The amalgamation of analyses from each jurisdiction involved comparison of logistic regression effect estimates. Analysis was undertaken using both SPSS® (Statistical Package for the Social Sciences) and SAS® (Statistical Analysis System) in different jurisdictions. Amalgamation of results is presented using forest plots (Fig. 1).

Ethics approval was granted in Alberta by the University of Calgary Conjoint Health Research Ethics Board, Nova Scotia by the IWK Research Ethics Board, and in BC by the UBC Behavioural Research Ethics Board.

## Results

The total number of singleton births included in the study period April 1st 2003 to March 31st 2008 is 150,797 of which 70,037 occurred in Alberta, 61,991 in BC, and 18,769 in Nova Scotia. The number of rural catchments (as of March 31st 2008) in Alberta is 107, 106 in BC, and 9 in Nova Scotia. Table 2 provides an overview for each jurisdiction, of the number of mothers and catchments in each level of service. Table 3 provides a summary of maternal characteristics by jurisdiction.

**Table 2:** Number of mothers and catchments in each service level ^a^

Service level	Alberta (*n* = 70,037)	Alberta Number of catchments in each service level	British Columbia (*n* = 61,991)	British Columbia number of catchments in each service level	Nova Scotia (*n* = 18,769)	Nova Scotia number of catchments in each service level	Total (*n* = 150,797)
1	322	4	601	14			955
2	1339	11	623	20	99	^	2070
3	3082	34	1892	23	1772	^	7024
4	7126	17	2976	11			14947
5	22666	30	6814	13	993	2	33000
6	4463	4	7206	7			17022
7	302	1	2778	2	440	1	3543
8	30737	6	39101	16	15465	6	85548

^a^Dataset excludes multiples, congenital anomalies, planned homebirths, and accidental out of hospital births ^ In Nova Scotia actual residence by address data was available so travel time calculations were made for individual women, rather than entire catchments

**Table 3:** Population characteristics by jurisdiction (2003/2004 to 2007/2008)

Characteristics		No. (%) of women from AB	No. (%) of women from BC	No. (%) of women from NS
		*n* = 70,037	*n* = 61,991	*n* = 18,769
Age	<18	1618 (2.3)	1256 (2.0)	413 (2.2)
	>35	5127 (7.3)	8866 (14.3)	2387 (12.7)
Multiparious		41730 (59.6)	35089 (56.6)	10656 (56.8)
Prior stillbirth		756 (1.1)	483 (0.8)	145 (0.8)
Prior neonatal death		393 (0.6)	285 (0.5)	63 (0.3)
Hypertension (pre-existing & gestational)		3754 (5.4)	3581 (5.8)	1439 (7.7)
Diabetes (pre-existing & gestational)		2325 (3.3)	2151 (3.5)	877 (4.7)

### Neonatal outcomes

In Tables 4, 5, 6, and 7, category 8 (maternity services provided by obstetricians) presents the adjusted odds ratios for key outcome variables in the three jurisdictions. Figure 1 presents the amalgamated forest plots for all three jurisdictions across all four outcomes. Perinatal mortality is highest in communities that are greater than 4 h from maternity services. Rates of prematurity at less than 37 weeks gestation are higher for rural women without local access to services while rates of very low birth weight (less than 1500 g) are not significantly different across service levels.

**Table 4:** Adjusted odds ratio for perinatal mortality^a^ by level of service, by jurisdiction

	Alberta		British Columbia		Nova Scotia	
Service level	Adjusted OR^b^ (95 % CI) (*n* = 66713)	*N*	Adjusted OR^b^ (95 % CI) (*n* = 61,991)	*N*	Adjusted OR^b^ (95 % CI) (*n* = 18,769)	*N*
1	1.40 (0.44, 4.39)	310	2.84 (1.58, 5.10)	601		
2	1.35 (0.77, 2.38)	1297	1.33 (0.59, 3.01)	623	N/A	99
3	1.50 (1.03, 2.18)	2940	0.79 (0.43, 1.45)	1892	0.66 (0.38, 1.14)	1772
4	1.23 (0.92, 1.64)	6750	1.12 (0.73, 1.70)	2976		
5	1.12 (0.91, 1.36)	21362	1.07 (0.79, 1.44)	6814	0.82 (0.38, 1.78)	993
6	0.88 (0.58, 1.32)	3884	1.07 (0.80, 1.42)	7206		
7	1.53 (0.49, 4.83)	264	0.96 (0.61, 1.51)	2778	0.60 (0.23, 1.65)	440
8	1.0	29906	1.0	39101	1.0	15465

^a^Perinatal mortality = stillbirths + neonatal deaths up to 7 days

^b^Adjusted for maternal age (<18, >35), parity, previous C-section, prior neonatal death, prior still birth, diabetes (existing & gestational), hypertension (existing & gestational)

**Table 5:** Adjusted odds ratio of prematurity < 37 weeks by level of service, by jurisdiction (excluding stillbirths)

	Alberta		British Columbia		Nova Scotia	
Service Level	Adjusted OR^a^ (95 % CI) (*n* = 66215)	*N*	Adjusted OR^a^ (95 % CI) (*n* = 61,991)	*N*	Adjusted OR^a^ (95 % CI) (*n* = 18,769)	*N*
1	1.22 (0.78, 1.91)	308	1.31 (1.00, 1.72)	601		
2	1.32 (1.06, 1.63)	1286	1.04 (0.78, 1.40)	623	0.86 (0.40, 1.87)	99
3	1.17 (1.00, 1.36)	2912	1.18 (1.00, 1.39)	1892	1.14 (0.92, 1.40)	1772
4	1.07 (0.96, 1.20)	6691	0.80 (0.68, 0.93)	2976		
5	1.08 (1.01, 1.17)	21194	0.87 (0.78, 0.96)	6814	0.95 (0.74, 1.23)	993
6	1.23 (1.08, 1.40)	3852	0.95 (0.86, 1.05)	7206		
7	1.26 (0.79, 2.03)	262	0.82 (0.70, 0.96)	2778	1.32 (0.86, 2.03)	440
8	1.0	29710	1.0	39101	1.0	15465

^1^Adjusted for maternal age (<18, >35), parity, previous C-section, prior neonatal death, prior still birth, diabetes (existing & gestational), hypertension (existing & gestational)

**Table 6:** Adjusted odds ratio of low birth weight < 1500 g by level of service, by jurisdiction

	Alberta		British Columbia		Nova Scotia	
Service level	Adjusted OR^a^ (95 % CI) (*n* = 66192)	*N*	Adjusted OR^a^ (95 % CI) (*n* = 62,894)	*N*	Adjusted OR^a^ (95 % CI) (*n* = 18,679)	*N*
1	0.98 (0.24, 3.99)	308	0.37 (0.52, 2.65)	592		
2	1.73 (1.01, 2.94)	1286	0.69 (0.17, 2.80)	617	0.60 (0.08, 4.39)	99
3	1.15 (0.75, 1.76)	2908	0.91 (0.45, 1.85)	1882	1.37 (0.69, 2.72)	1772
4	1.10 (0.81, 1.48)	6681	0.72 (0.38, 1.36)	2953		
5	0.98 (0.80, 1.21)	21162	0.89 (0.59, 1.32)	6772	0.72 (0.37, 1.39)	993
6	0.93 (0.62, 1.38)	3856	0.83 (0.56, 1.24)	7162		
7	1.63 (0.51, 5.14)	262	0.91 (0.51, 1.64)	2763	0.56 (0.24, 1.30)	440
8	1.0	29729	1.0	38867	1.0	15462

^a^Adjusted for maternal age (<18, >35), parity, previous C-section, prior neonatal death, prior still birth, diabetes (existing & gestational), hypertension (existing & gestational)

**Table 7:** Adjusted odds ratio of caesarean section by level of service, by jurisdiction

	Alberta		British Columbia		Nova Scotia	
Service level	Adjusted OR^a^ (95 % CI) (*n* = 66713)	*N*	Adjusted OR^a^ (95 % CI) (*n* = 63,277)	*N*	Adjusted OR^a^ (95 % CI) (*n* = 18,766)	*N*
1	0.64 (0.48, 0.87)	310	0.70 (0.57, 0.85)	601		
2	0.67 (0.58, 0.77)	1297	0.74 (0.61, 0.90)	623	0.67 (0.40, 1.10)	99
3	0.86 (0.78, 0.94)	2940	0.92 (0.83, 1.03)	1892	0.87 (0.77, 0.98)	1772
4	0.81 (0.76, 0.86)	6750	0.84 (0.78, 0.92)	2976		
5	1.07 (1.03, 1.12)	21362	0.91 (0.86, 0.96)	6814	1.08 (0.93, 1.24)	993
6	0.91 (0.84, 0.99)	3884	0.95 (0.89, 1.00)	7206		
7	1.29 (0.99, 1.69)	264	1.19 (1.09, 1.29)	2778	0.91 (0.73, 1.14)	440
8	1.0	29906	1.0	39101	1.0	15462

^a^Adjusted for maternal age (<18, >35), parity, previous C-section, prior neonatal death, prior still birth, diabetes (existing & gestational), hypertension (existing & gestational)

### Intervention rates

Caesarean section rates are highest in communities served by general surgical models. Lack of local access to surgical care is associated with lower caesarean section rates, both for communities from which women have to travel to access maternity services and for communities with local intrapartum services without caesarean section capabilities (Table 7, Fig. 1). There is a trend towards a lower rate of intervention the farther from services women are.

## Discussion

This multi-jurisdictional Canadian study strengthens conclusions from previous research at the provincial or regional level. It demonstrates that lack of local access to intrapartum maternity services is associated with poorer neonatal outcomes [25]. It raises the question of whether centralization of maternity services in rural referral centres is ultimately an effective strategy for achieving best perinatal outcomes for rural families. Communities that provide local elective intrapartum services without local access to Cesarean Section are able to achieve better outcomes than communities without local services. This strongly suggests that we should stop closing rural maternity services and do what we can to reverse previous closures. Communities that rely on general surgical backup to provide caesarean section services have higher rates of intervention than communities with surgical services staffed by general practitioners (GPs) with enhanced surgical skills or mixed provider models. This finding supports the scaling up of programs that train Canadian graduates and attract International Medical Graduates with enhanced surgical skills to work in rural Canada.

The limitations of this study are the relatively small number of outcomes that we could examine multi-jurisdictionally due to differing variable definition and data collection practices in the three jurisdictions studied, let alone other Canadian provinces. There were also some differences between the jurisdictions e.g., Tables 5 and 7, level 5, which should prompt a further local analysis. While potential confounders related to perinatal outcomes were considered in the regression modelling, we were unable to consider the effects of ethnicity and socioeconomic status across these diverse regions as this data is not available. A secondary study is underway in Alberta to examine these effects on Alberta rural perinatal outcomes. We were also unable to adjust for more than one singleton birth to individual mothers within the study period due to the lack of availability of data linkages. We estimate that this is likely to have negligible influence on the effect sizes.

This study has had to overcome significant barriers to the amalgamation of results from provincial data repositories. While patients’ confidential information needs to be protected, the barriers thus justified are detrimental to the larger goals of studying and addressing problems on a national scale in accessing optimal health care services. If we are going to move forward collectively, scale up successful innovations and learn from each other, we need National and Provincial health policy designed to facilitate this collaborative work.

## Conclusion

Composite analysis of data from three Canadian provincial jurisdictions provides the strongest evidence to date demonstrating that we need to sustain small community maternity services with and without caesarean section capability. Rural caesarean section services staffed by GPs with enhanced surgical skills provide safe care and should be supported.
